# Glioblastoma Multiforme Stem Cell Cycle Arrest by Alkylaminophenol through the Modulation of EGFR and CSC Signaling Pathways

**DOI:** 10.3390/cells9030681

**Published:** 2020-03-10

**Authors:** Phuong Doan, Aliyu Musa, Akshaya Murugesan, Vili Sipilä, Nuno R. Candeias, Frank Emmert-Streib, Pekka Ruusuvuori, Kirsi Granberg, Olli Yli-Harja, Meenakshisundaram Kandhavelu

**Affiliations:** 1 Molecular Signaling Lab, Faculty of Medicine and Health Technology, Tampere University, P.O. Box 553, 33101 Tampere, Finland; phuong.doan@tuni.fi (P.D.); aliyu.musa@tuni.fi (A.M.); akshaya.murugesan@tuni.fi (A.M.); vili.sipila@tuni.fi (V.S.); 2 BioMediTech Institute and Faculty of Medicine and Health Technology, Tampere University, Arvo Ylpön katu 34, 33520 Tampere, Finland; pekka.ruusuvuori@tuni.fi (P.R.); kirsi.granberg@tuni.fi (K.G.); olli.yli-harja@tuni.fi (O.Y.-H.); 3 Science Center, Tampere University Hospital, Arvo Ylpön katu 34, 33520 Tampere, Finland; 4 Predictive Society and Data Analytics Lab, Faculty of Information Technology and Communication Sciences, Tampere University, 33101 Tampere, Finland; frank.emmert-streib@tuni.fi; 5 Department of Biotechnology, Lady Doak College, Thallakulam, Madurai 625002, India; 6 LAQV-REQUIMTE, Department of Chemistry, University of Aveiro, 3810-193 Aveiro, Portugal; ncandeias@ua.pt; 7 Institute of Biosciences and Medical Technology, 33101 Tampere, Finland; 8 Computational Systems Biology Group, Faculty of Medicine and Health Technology, Tampere University, P.O. Box 553, 33101 Tampere, Finland; 9 Institute for Systems Biology, 1441N 34th Street, Seattle, WA 98103-8904, USA

**Keywords:** GBM stem cells, non-stem cancer cells, resistance population, cell cycle arrest, alkylaminophenol and cell death

## Abstract

Cancer stem cells (CSCs), a small subpopulation of cells existing in the tumor microenvironment promoting cell proliferation and growth. Targeting the stemness of the CSC population would offer a vital therapeutic opportunity. 3,4-Dihydroquinolin-1(2*H*)-yl)(*p*-tolyl)methyl)phenol (THTMP), a small synthetic phenol compound, is proposed to play a significant role in controlling the CSC proliferation and survival. We assessed the potential therapeutic effects of THTMP on glioblastoma multiforme (GBM) and its underlying mechanism in various signaling pathways. To fully comprehend the effect of THTMP on the CSCs, CD133^+^ GBM stem cell (GSC) and CD133^-^ GBM Non-stem cancer cells (NSCC) population from LN229 and SNB19 cell lines was used. Cell cycle arrest, apoptosis assay and transcriptome analysis were performed for individual cell population. THTMP strongly inhibited NSCC and in a subtle way for GSC in a time-dependent manner and inhibit the resistance variants better than that of temozolomide (TMZ). THTMP arrest the CSC cell population at both G1/S and G2/M phase and induce ROS-mediated apoptosis. Gene expression profiling characterize THTMP as an inhibitor of the p53 signaling pathway causing DNA damage and cell cycle arrest in CSC population. We show that the THTMP majorly affects the EGFR and CSC signaling pathways. Specifically, modulation of key genes involved in Wnt, Notch and Hedgehog, revealed the significant role of THTMP in disrupting the CSCs’ stemness and functions. Moreover, THTMP inhibited cell growth, proliferation and metastasis of multiple mesenchymal patient-tissue derived GBM-cell lines. THTMP arrests GBM stem cell cycle through the modulation of EGFR and CSC signaling pathways.

## 1. Introduction

Glioblastoma multiforme or simply glioblastoma (GBM) is the most common malignant primary tumor. GBM is a grade IV astrocytoma that accounts for up to 60% of gliomas and carries the worst prognosis of all the cancers [1]. The current treatment of GBM using an alkylating agent, temozolomide (TMZ) combined with radiotherapy, shows their transient effect in only a few subsets of patients [2,3,4]. Hence, there is an urgent need for exploring effective therapeutics for GBM. To achieve this goal, many efforts have been made in understanding the complicated mechanisms that control GBM growth. 

The initiation and progression of cancer is governed by a small subset of tumor-initiating cells termed cancer stem cells (CSC), whereas in GBM, GBM stem cells (GSCs) have a similar phenotype to the normal neural stem cells [5]. CSCs accounts for a rare fraction of a certain tumor that is responsible for tumor characteristics such as invasion, metastasis and relapse. The self-renewal ability of those differentiated cells allows them to become resistant to the current modern medicine involving radiation and chemotherapy [6]. Hence, research centering to hamper the activation of CSC via modulation of signaling pathway is much appreciated. Molecules such as CD133, CD44, ABCG2 and ALDH are believed to function as a biomarkers in some kind of CSCs [7,8,9,10]. Furthermore, several signaling pathway have been associated in the self-renewal behavior of cancer stem cells including Wingless-Int (Wnt), Notch and Hedgehog (Hh) pathways [11,12,13,14]. In addition to the CSC signaling pathways, EGFR signaling is also reported as a primary contributor to GBM initiation and progression. Oncogenic role of EGFR driving to GBM tumorigenesis has been validated both in in vivo and in vitro models [15,16,17,18,19]. Thus, targeting important downstream signaling pathways in CSC is important for identifying novel GBM therapy.

In the past decades, several phenolic compounds have been approved by FDA as anticancer agent including vincristine for small-cell lung cancer [20], paclitaxel for metastatic breast cancer [21], omacetaxine for chronic myeloid leukemia [22] etc. Among various phenolic compounds, alkylaminophenol moiety is found in some FDA-approved drugs such as amodiaquine and hycamtin (topotecan) is used as a promising chemotherapeutic agents [23,24]. Earlier reports from our research group have shown that several alkylaminophenols functions as an inducer of apoptosis on osteosarcoma and GBM [25,26,27,28]. Although anticancer activity of phenolic derivatives has been well reported on various cancers, their effect on GBM is not well investigated. Hence, the present study is aimed at evaluating the effect of alkylaminophenols on GSC and non-stem cancer cells (NSCC) derived from two different GBM cell lines (LN229 and SNB19). The profound investigation on the cellular mechanisms has been performed, which revealed the cytotoxic effect of alkylaminophenols on GSC. The multiple patient-derived GBM cell lines were also used to further confirm the potential of the compound as an effective chemotherapeutic agent for GBM.

## 2. Materials and Methods

### 2.1. Chemical Preparation

**Figure 1:**
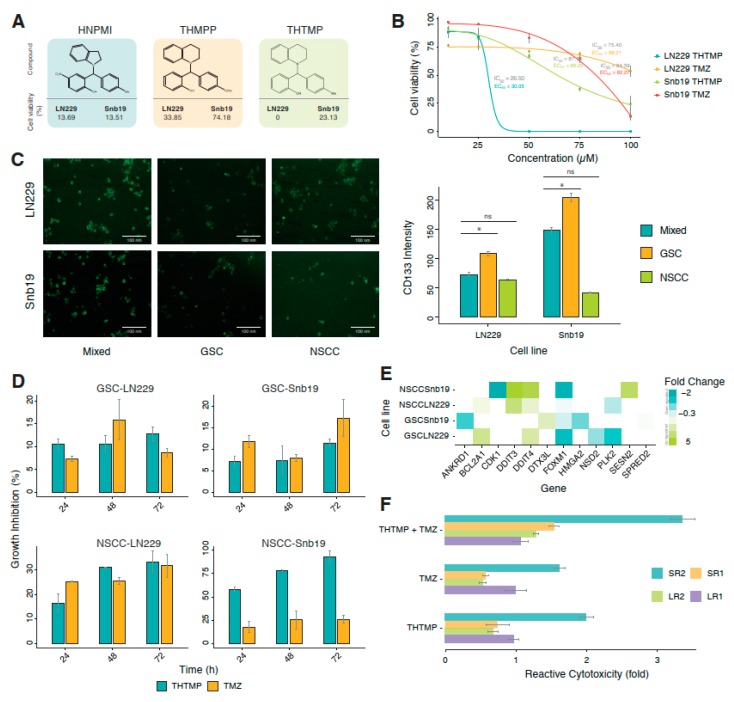
Effect of 3,4-Dihydroquinolin-1(2*H*)-yl)(*p*-tolyl)methyl)phenol (THTMP) treatment on cell survival in GBM cells. (**A**) Molecular structure of three tested alkylaminophenols (HNPMI, THMPP and THTMP) and % cell viability of those compounds on LN229 and SNB19 cell lines at 100 µM concentration. (**B**) Percentage of cell viability for LN229 and SNB19 cell lines upon treatment with THTMP/TMZ in the dilution series from 10 µM to 100 µM. (**C**) Representative image and intensity of CD133 on GSC, NSCC and mixed population of LN229 and SNB19. (**D**) Time-dependent effect of THTMP on GSC and NSCC cells. The results were normalized to DMSO control. One-way ANOVA was conducted (*P* < 0.05) to determine the statistical significance in all the conditions compared to DMSO control. (**E**) The top DEGs involved in DNA damage on GSC-LN229, GSC-SNB19, NSCC-LN229 and NSCC-SNB19. The DEGs were color coded corresponding to the up- and down- expressed genes. (**F**) Relative cytotoxicity of resistant variants, LR1, LR2 (derived from GSC-LN229) and SR1, SR2 (derived from GSC-SNB19) to THTMP and/or TMZ. The results were normalized to DMSO control. One-way ANOVA was done (*P* < 0.05) to determine the statistical significance in all the conditions compared to DMSO control. All experiments were performed with N = 5. * *p* < 0.05, ns—non significant.

Synthesis and spectral characterization of compounds HNPMI [25], THMPP [26] and THTMP [29] (Figure 1A) were previously reported. These compounds and temozolomide (Sigma-Aldrich, St. Louis, MO, USA) were dissolved in dimethyl sulphoxide (DMSO, Sigma-Aldrich) to obtain a stock solution of 100 mM. Intermediate dilutions were prepared using stock solution.

### 2.2. Cell Viability and Heterogeneity from Dose-Response Curves

The cell viability assay was performed to determine the inhibitory effect of the compounds against the growth of GBM cells, LN229 and SNB19. The cell lines were gifted by Dr.Kirsi Granberg, Faculty of Medicine and Health Technology, Tampere, Finland). LN229 was originated from a patient with right frontal parieto-occipital glioblastoma with mutated p53 and homozygous deletions in the p16 and p14ARF tumor suppressor genes. SNB19 was derived from a patient with the left parietooccipital glioblastoma tumor. The cells were cultured in Dulbecco’s Modified Eagle Medium (DMEM) full medium (DMEM, 10% FBS, 0.1 mg/mL streptomycin, 100 U/mL penicillin, and 0.025 mg/mL amphotericin B). The concentrations of 100 µM, 75 µM, 50 µM, 25 µM, and 10 µM of each compound (HNPMI, THMPP, and THTMP) were used to determine the cell viability. After 24 h exposure, the cells were collected by centrifugation at 3000 rpm for 5 min. Cell viability was determined by trypan blue and Countess II FL Automated Cell Counter (ThermoFisher Scientific, Carlsbad, CA, USA). Half-maximal inhibitory concentration (IC_50_) values were calculated based on the sigmoidal dose-response curves, which were generated in the Matlab 2013a software using logistic function. Then, the half-maximal effective concentration (EC_50_) of cell death inducing compounds were calculated from these dose-response curves as suggested previously [30] by using the following formula:

(1) $${\mathrm{EC}}_{50}=Yb+\frac{Y_{T}-Y_{B}}{1+10^{\left(LogEC50-X\right)*HS}}$$

where, *Y*_B_ is the *Y* value at the bottom plateau, *Y*_T_ is the *Y* value at the top plateau, *LogEC*_50_ is the *X* value when the response is halfway between bottom plateau and top plateau, and *HS* is the Hill coefficient [31].

### 2.3. Isolation of Glioblastoma Stem Cells (GSC) and Non-Stem Cancer Cell (NSCC) and Cell Culture

In GBM, CD133 has been accepted as a marker for CSCs which was isolated using CD133 MicroBead Kit (Miltenyi Biotec, Lund, Sweden). The cells containing CD133 enrichment are classified as GBM stem cells (GSC) and the cells containing CD133 depletion are defined as GBM non-stem cancer cells (NSCC). The procedure for the isolation of the GSC and NSCC was followed as instructed by the manufacturer. Briefly, after harvesting the cells, 300 µL of buffer was added to 1 × 10^8^ total cells. Then, 100 µL of the FcR blocking reagent and CD133 MicroBeads were added into the buffer containing cells. The mixture was mixed, incubated for 30 min at 4 °C and the cells were then washed with buffer to remove the reagents. The cells are subjected for magnetic separation using columns supplied with the kit and MACS separator.

In this study, we used LN229 and SNB19 GBM cells for GSC and NSCC isolation. GSC-LN229 and GSC-SNB19 cells were cultured in StemPro hESC SFM medium (Life Technologies, Pleasanton, CA, USA) while NSCC-LN229 and NSCC-SNB19 cells were cultured in DMEM full medium. The cells were maintained at 37 °C in a humidified atmosphere containing 5% CO_2_. All of the components of the cell culture were purchased from Sigma-Aldrich.

### 2.4. Pharmacodynamics Study

The time-dependent study was performed using IC_50_ concentration of THTMP on LN229 and SNB19 as described previously [28]. GSC and NSCC are treated with THTMP for 24, 48 and 72 h. Treated cells were collected using centrifugation at 3000 rpm for 10 min. Number of live and dead cells were determined using trypan blue solution and Countess II FL Automated Cell Counter (ThermoFisher Scientific). Inhibition percentage was calculated using Equation (2). Biological and technical replicates were conducted for each condition. TMZ and DMSO vehicle were used as positive and negative control, respectively.

(2) $$\mathrm{Inhibition}\;(\%)=\frac{\mathrm{Mean}\;\mathrm{No}.\;\mathrm{of}\;\mathrm{untreated}\;\mathrm{cells}\;(\mathrm{DMSO}\;\mathrm{control})-\mathrm{Mean}\;\mathrm{No}.\;\mathrm{of}\;\mathrm{treated}\;\mathrm{cells}}{\mathrm{Mean}\;\mathrm{No}.\;\mathrm{of}\;\mathrm{untreated}\;\mathrm{cells}\;(\mathrm{DMSO}\;\mathrm{control})}\times 100$$

### 2.5. Resistance Variants

The response rate to current chemotherapies for cancer depends on the availability of various chemotherapy regimens. However, multidrug resistance (MDR) still develops nearly in all patients with cancers and leads to chemotherapy failure [32,33]. We tested the ability of THTMP and TMZ on creating drug-resistant tumor cells. The THTMP-resistant and TMZ-resistant variants of GSC-LN229 and GSC-SNB19 cells were obtained by exposing the cells to THTMP/TMZ as follows. GSC-LN229 and GSC-SNB19 cells were cultured at an initial density of 5 × 10^5^ cells per 25-cm^2^ flask containing 10 mL medium for 3 days. The cells were then treated with 5 µM of compound (THTMP/TMZ) for 24 h. The cells were then cultured in the compound-free medium for 2 weeks to recover the cell density. The above treatment was repeated five times. The variants that survived THTMP and TMZ exposure were designated as R1 and R2, respectively. After obtaining R1 and R2, cytotoxicity assay was performed by exposing the cells to the IC_50_ concentration of THTMP and/or TMZ for 24 h. The cell viability assay and the cell growth inhibition were carried out as described in Section 2.4.

### 2.6. Illumina Sequencing, RNA-Seq Data and Gene Ontology Analysis

To perform Illumina sequencing, RNA samples were isolated from GSC and NSCC. GSC-LN229, NSCC-LN229, GSC-SNB19 and NSCC-SNB19 cells were cultured as described in Section 2.3. The cells were then treated with the IC_50_ concentration of THTMP/TMZ for 24 h. RNA of treated GSC and NSCC was isolated using GeneJET RNA Purification Kit (ThermoFisher Scientific, Waltham, MA, USA) following the manufacturer’s instruction. Whole transcriptome sequencing of the total RNA samples of GSC-LN229, NSCC-LN229, GSC-SNB19 and NSCC-SNB19 cells (including triplicates of THTMP treated, TMZ treated and untreated samples) were performed by the Biomedicum Functional Genomics Unit (FuGU, University of Helsinki, Helsinki, Finland) using Illumina NextSeq 500. The sequencing produced data in bcl format, which was converted into FASTQ file format. The RNA-seq data analysis pipeline, gene ontology and pathway analysis was performed as described previously [28]. In this analysis, only pathways and GO term with *p*-value <0.05 and fold change of 1.5 were set as cutoff values.

### 2.7. Cell Cycle Analysis

GSC-LN229, NSCC-LN229, GSC-SNB19 and NSSC-SNB19 cells were cultured in 6 well-plate at an initial density of 5 × 10^5^ cells/well. The cells were treated with the IC_50_ concentration of the compounds (THTMP or TMZ) for 24 h. The cells were then harvested, washed in ice cold PBS and fixed in 70% ice-cold ethanol for 30 min at 4 °C. After washing in cold PBS, the cells were suspended in 200 μL PBS containing 20 µg/mL propidium iodide (PI), 0.2 mg/mL RNase and 0.1% triton X-100 and incubated for 30 min at 37 °C. Fluorescence images were captured using EVOS imaging system (ThermoFisher Scientific, Waltham, MA, USA) with 10× objective magnification. The cell cycle phases were analyzed using CellProfiler [34,35].

### 2.8. Annexin V-Fluorescein Isothiocyanate (FITC)/Propidium Iodide (PI) Labelling (V-FITC/PI) Double Staining Assay

Apoptosis induction assay was performed using Dead Cell Apoptosis Kit with Annexin V–fluorescein isothiocyanate (FITC) and propidium iodide (PI) (ThermoFisher Scientific, Waltham, MA, USA) followed by the manufacturer’s protocol. Briefly, GSC-LN229, NSCC-LN229, GSC-SNB19 and NSSC-SNB19 cells were seeded in 6 well-plate with the initial density of 5 × 10^5^ cells/well. The cells were treated with the IC_50_ concentration of THTMP for 24 h, and then harvested and washed in ice-cold PBS. The cell pellets were then resuspended in 1X annexin-binding buffer provided along with the kit. Then, 5 μL of FITC conjugated Annexin V and 1 μL of the 100 μg/mL PI were added to 100 μL of the cell suspension. The cells were incubated at RT for 15 min prior to the fluorescence measurements. The image acquisition was done by using EVOS imaging system (ThermoFisher Scientific, Waltham, MA, USA) with 20× objective magnification. The positive control (TMZ) and negative control (DMSO) were also included in the study.

### 2.9. Detection of Intracellular Reactive Oxygen Species (ROS) and Caspase 3/7 Activity

GSC-LN229, NSCC-LN229, GSC-SNB19 and NSCC-SNB19 cells were cultured overnight in the appropriate culture conditions. The cells were then treated with the IC_50_ concentration of THTMP/TMZ for 5 h. Cells were harvested by centrifugation at 3000 rpm for 10 min. For ROS detection, the cells were incubated with 2 μM 2′,7′-dichlorodihydrofluorescein diacetate (H2DCFDA) (Sigma-Aldrich), for 30 min at cell culture condition. The cells were then washed and recovered in pre-warmed complete medium for 20 min prior to the fluorescence measurement at the excitation wavelength of 485 nm and emission wavelength of 538 nm by Fluoroskan Ascent FL (Thermo Labsystems; St. Louis, MO, USA). DMSO and hydrogen peroxide (200 µM) were used as the negative and positive controls. For caspase 3/7 detection, we used Caspase-Glo® 3/7 Assay kit (Promega, Madison, WI, USA) following the standard protocol of the manufacturer. Caspase-Glo reagent was added to the plate containing treated cell, untreated cell, blank or TMZ with the ratio of 1:1 (*v/v*). After that, the cells were gently mixed and incubated for 1 h. The luminescence was measured using Fluoroskan Ascent FL (Thermo Labsystems).

The fold increase in ROS production and caspase 3/7 were calculated using Equation (3):

(3) $$Fold\;increase=\frac{F_{test}-F_{blank}}{F_{control}-F_{blank}}$$

where, *F_test_* is the fluorescence/luminescence readings from the treated wells, *F_control_* is the fluorescence/luminescence readings from the untreated wells, and *F_blank_* is the fluorescence/luminescence readings from the unstained wells.

### 2.10. In Vitro Cytotoxicity in Patient-Derived (GBM) Cells

Three cell lines from low-passage patient-derived primary GBMs (MMK1, RN1 and PB1), display the phenotype of GBM, were a gift from Dr. Brett Stringer (QIMR Berghofer, Medical Research Institute, QLD, Australia). The generation of these low-passage primary patients’ GBMs was done by isolating the patient’s tumor, which was approved by the human ethics committee of the Queensland Institute of Medical Research and Royal Brisbane and Women’s Hospital (ethical approval number: P3420, HREC/17/QRBW/577 Novel Therapies for Brain Cancer) [36]. These cells were then cultured in the serum-free medium using 1% matrigel-coated flasks, as previously described [37]. The cells were maintained in an incubator at 37 °C in humidified air with 5% CO_2_. MMK1, RN1 and PB1 cell lines were plated in 12-well plates (1 × 10^5^ cells/well), treated with THTMP/TMZ (100 and 10 µM) for 24 h. The cell viability assay and the cell growth inhibition were carried out as described in the Section 2.4.

### 2.11. Wound Healing Assay on Patient-Derived (GBM) Cells

Initial density of 3 × 10^5^ cells/well was plated in 12-well plate. MMK1, RN1 and PB1 cells were cultured as described in Section 2.10 until reaching the monolayer confluency. A scratch was made in each well using a thin tip. The edges were smoothened and washed with the phosphate-buffered saline (PBS). The cells were then treated with 30 µM of THTMP/TMZ. The scratched area was visualized under a light microscope every 2 h for a total period of 10 h.

### 2.12. Statistical Analysis

The following experiments such as, cell viability assay, pharmacodynamics study, Illumina sequencing, cell cycle progression, annexin V-FITC/PI staining, ROS and caspase assay, in-vitro cytotoxicity in patient derived GBM cells and wound healing assay, were conducted with five biological repeats and technical repeats. The data were shown as means ± S.D and analysed using IBM SPSS (Statistics for Windows version 20.0). For comparison between the tested groups, statistical significant differences were evaluated using the *t*-test. For comparison of more than two groups, statistical significance was determined using a one-way ANOVA test. *p* < 0.05 was considered statistically significant.

## 3. Results

### 3.1. Effect of Alkylaminophenol in Glioblastoma Multiforme (GBM) Cell Lines

A series of alkylaminophenols, HNPMI, THMPP and THTMP, were investigated for their induction of cell death on multiple GBM cancer cell lines. THTMP has the highest inhibitory effect on the GBM cells at a concentration of 100 µM (Figure 1A) compared with HNPMI and THMPP. Although these three compounds have shown promising cell growth inhibition on GBM cell lines, LN229 and SNB19, the presence of methyl group in the place of 4-OMe in THTMP improved the percentage of cytotoxicity. THTMP shows 0% and 23.13% cell viability on LN229 and SNB19, respectively. HNPMI shows a similar effect on both GBM cell lines, whereas THMPP has varying cell death effect. The top lead compound, THTMP potentially inhibited the GBM cell proliferation in a dose-dependent manner (Figure 1B) in which the IC_50_ values were about 26.5 µM and 87.8 µM for LN229 and SNB19, respectively. We also previously reported that, THTMP has selectively inhibited the growth of GBM cells than the non-cancerous cells (Supplementary Figure S1) [28]. IC_50_ of positive drug control, TMZ on these two cell lines was also calculated to be 75.4 µM and 84.4 µM, respectively. Sigmoidal dose-response curve was used to calculate the EC_50._ The EC_50_ values of THTMP and TMZ for the respective cell lines were about 30.1 µM and 88.2 µM (LN229) as well as 69.3 µM and 82.3 µM (SNB19), respectively. Thus, the improved EC_50_ values were observed for THTMP than the TMZ on both the cell lines.

### 3.2. Glioblastoma Stem Cells (GSC) and Non-Stem Cancer Cell (NSCC) Show a Heterogeneous Sensitivity to 3,4-Dihydroquinolin-1(2H)-yl)(p-tolyl)methyl)phenol (THTMP) and Temozolomide (TMZ) Treatment in Time-Dependent Manner

There are evidences affirming that tumor-specific stem cell populations are the key contributor to the failure in chemotherapy and radiotherapy. The eradication of CSC population is the necessity that could support the therapies for the efficient reduction in the progression of the tumor. Thus, to pursue this insight, we have evaluated the efficacy of THTMP and TMZ on GSCs and NSCCs derived from LN229 and SNB19. The expression of CD133 in each population was tested using CD133 antibody. The intensity of CD133 was found to be higher in GSC than in the NSCC and its mixed population (Figure 1C). Also, the CD133 was found to be overexpressed in the GSC and mixed population of SNB19 than LN229.

In order to identify the effect of THTMP/TMZ on the GBM sub-population, GSC-LN229, GSC-SNB19, NSCC-LN229 and NSCC-LN229, we have examined the growth-inhibitory effect using the IC_50_ concentration after 24, 48 and 72 h of THTMP/TMZ exposure. GSCs and NSCCs responded heterogeneously on THTMP and TMZ exposure (Figure 1D). We noticed a gradual increase in the percentage of cell death upon THTMP treatment, while TMZ induced varying cell death in GSC population of both cell lines over the time. THTMP has higher growth inhibition than the TMZ in NSCC population of both cell lines. Time dependent treatment of THTMP has shown a considerable increase of cell death in NSCC than GSC. NSCC-SNB19 has cell death by 90%, whereas NSCC-LN229 has 32%, after 72 h of treatment.

To further explore the underlying mechanism on the effect of THTMP at 24 h post treatment, the differential expression of genes (DEGs) involved in the DNA damage was analysed. DEGs with two-fold changes or greater (*p* < 0.001) upon THTMP treatment were quantitatively analysed (Supplementary Tables S1–S4). Totally twelve DEGs associated with the DNA damage were listed (Figure 1E). *FOXM1* and *CDK1* overexpression induces carcinogenesis and disease progression in GBM. *FOXM1* upregulation regulates G2/M phase in cell cycle and P53 signaling pathway [38]. Upon THTMP treatment, *FOXM1* was downregulated in GSC and NSCC populations of both cell lines. Besides *FOXM1*, we also noticed the downregulation of *p53* target genes, *PLK2* in GSC-LN229, and NSCC-LN229. Thus, THTMP can target p53 signaling pathway in GBM cells.

Upon THTMP treatment, more DEGs were found in NSCC than in GSC population. This may be due to the ability of GSC to resist the effect upon THTMP on due course of treatment. Since the development of resistant GSC cells prevented them from being subjective for THTMP and/or TMZ treatment, we investigated the mechanism of alteration in the sensitivity of resistant variants. GSC resistant variants were developed upon treatment with THTMP and/or TMZ as described in the method section. On treatment with THTMP, the cytotoxicity effect of GSC-LN229 derived cells, LR1 and LR2 were 1.0 and 0.7-fold higher than the respective parent cells, whereas GSC-SNB19 derived cells SR1 and SR2 were 0.8 and 2.1-fold higher, respectively. In parallel, the cytotoxic effect of TMZ and combination of THTMP/TMZ on the resistant variants was also analysed. These results substantiated the above findings that there was higher cytotoxic potential of THTMP than the TMZ. Thus, synergy of THTMP with TMZ on both GSC variants showed higher cytotoxicity (i.e., % of cell death in THTMP treated cells <THTMP + TMZ; % of cell death in TMZ treated cells <THTMP + TMZ).

### 3.3. THTMP Triggers Cell Cycle Arrest in GSCs and NSCCs

**Figure 2:**
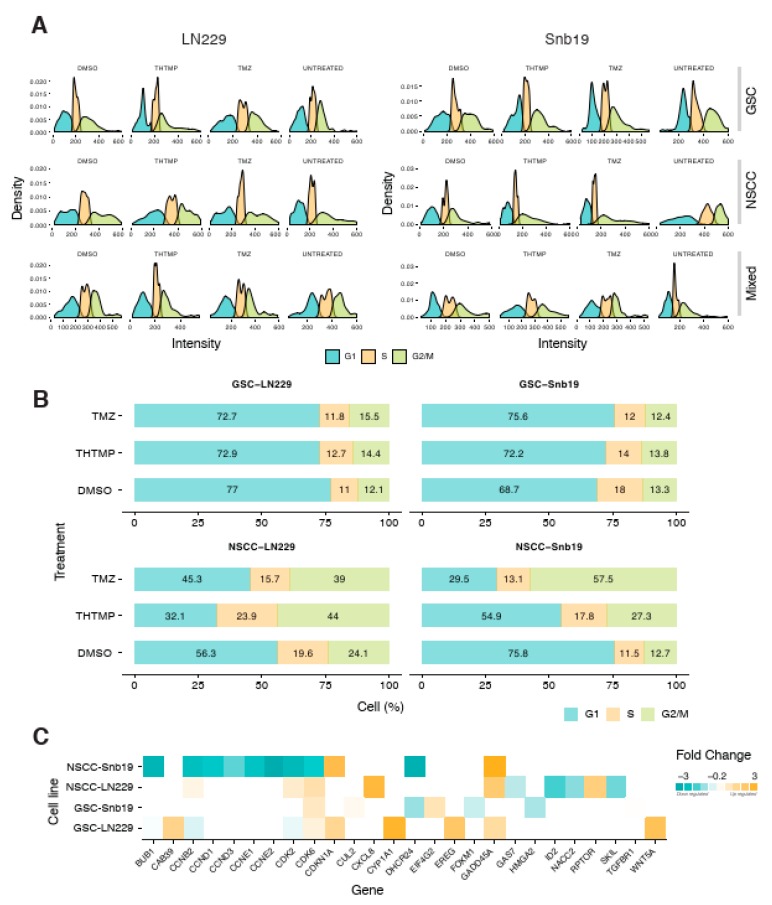
THTMP triggers GSC and NSCC cell cycle arrest. (**A**) Graphs represented the distribution of cells in different phases of cell cycle (**B**) The bar diagram representing the percentage of total cells in different cell cycle phases treated with THTMP/TMZ. (**C**) The top DEGs involved in the cell cycle in GSC and NSCC population. The DEGs were color coded corresponding to the up- and down- expressed genes. Five biological and technical repeats were used in cell cycle and gene expression analysis. All experiments were performed with N = 5.

To gain more insights into the effect of THTMP/TMZ on the cell viability and cell proliferation, we examined the cell cycle phases in GSCs and NSCCs. Cells in G1, S and G2/M phase were separated based on the linear fluorescence intensity of propidium iodide stain. The large initial peak (left) represents the cells in G1, the intervening area represents cells in S phase and the final tail/small peak (right) represents cells in G2/M phase. THTMP treatment induced the cell cycle arrest at G1/S phase in both GSC-LN229 and GSC-SNB19 cells (Figure 2A). This inference is made by comparing the proportion of cells present in each phase. As shown in Figure 2A, we could observe the majority of cells in G1 phase, with significant reduction of cells in S phase and with further loss in G2/M phase. Subsequently, cell cycle analysis of NSCC-LN229 revealed that most of the cells arrested in G2/M phase, whereas SNB19 arrest happens in G1/S phase of NSCC-SNB19 cells.

The arrest in the different phases of the cell cycle is possible due to the presence of heterogeneous cell populations in NSCCs. As shown in Figure 2B, upon THTMP treatment, both GSC-LN229 and GSC-SNB19 have ~70% of cells in G1 phase while ~15% in S phase and G2/M phase. Most of the cells were arrested in G1 phase, suggesting the smaller number of cells entering into the S phase and G2/M phase. Although the same pattern was observed in DMSO conditions of NSCC of both cell lines, the percentage of NSCCs found to be fluctuating on THTMP treated conditions.

THTMP treatment leads to 44%, 23.9% and 32.1% of cells in G2/M phase, S phase and G1 phase, respectively in NSCC-LN229, whilst 27.3%, 17.8% and 54.9% of the cells were observed in G2/M, S phase and G1 phase, respectively in NSCC-SNB19. Thus, NSCC-LN229 cells were arrested at G2/M phase and NSCC-SNB19 cells at G1/S phase.

The results obtained from cell cycle assay were substantiated by the transcriptome profiling. The THTMP could induce several biological processes involved in cell cycle pathways such as cell cycle G1/S phase transition, G1/S transition of mitotic cell cycle, mitotic cell cycle checkpoint, cell cycle DNA replication, cell cycle G2/M phase transition, G2/M transition of mitotic cell cycle, cell cycle arrest (Supplementary Tables S5–S8).

As shown in Figure 2C, number of genes were significantly enriched that corresponds to the cell cycle pathway. In NSCC-LN229, there occurs downregulation of *CCNB2* coding to cyclin B2, and an upregulation of *GADD45A* which is a downstream target gene of *p53* in G2 checkpoint. This suggests that NSCC-LN229 cells were arrested at G2/M phase. Meanwhile, NSCC-SNB19, GSC-LN229 and GSC-SNB19 showed the upregulation of *CDK2* and *CDK6* that are associated with G1/S arrest, whereas genes coding for cyclin D1, D2, E1, and E2 such as *CCND1*, *CCND2*, *CCNE1* and *CCNE2* respectively, were downregulated. These cyclins are essential for triggering and regulating G2/M transition in complex with *CDK1*. In addition, the most significant event observed was the downregulation of *BUB1* gene expression, which played vital role in G1/S transition of mitotic cells and DNA replication. Thus, transcriptome data revealed that, THTMP induced cell cycle arrest at G1/S or G2/M phase, which is explicitly specific for different cell line populations. Taken together the cell cycle arrest and gene expression analysis, THTMP could possibly inhibit the synthesis before they enter the synthesis phase or mitotic phase and thus potentially prevents cell proliferation.

### 3.4. THTMP Induces the Apoptosis via ROS and Caspase 3/7 Activation

**Figure 3:**
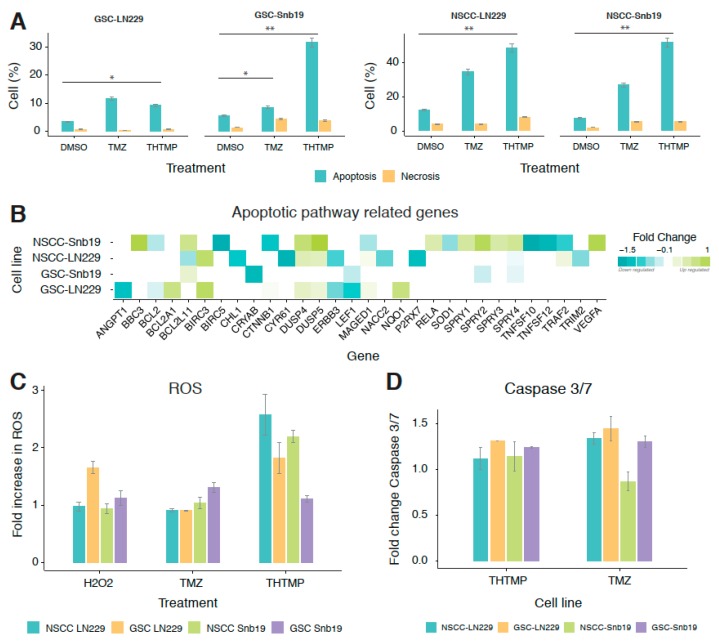
THTMP induces apoptosis in NSCCs and GSCs. (**A**) Percentage of apoptotic cells and necrotic cells upon THTMP/TMZ treatment at 24 h, stained with Annexin V-FITC/PI. (**B**) The top DEGs which are involved in apoptosis induction on GSCs and NSCCs. The DEGs were color coded that corresponds to the up- and down-expressed genes. (**C**) Effect of THTMP/TMZ on NSCCs and GSCs intracellular ROS production. (**D**) Activity of caspases 3/7 on NSCCs and GSCs in THTMP/TMZ treatment. Fold increase in ROS and caspases 3/7 activity of cells was calculated with triplicates for each condition. The data were normalized against DMSO control (**C**,**D**). * *p* < 0.05, ** *p* < 0.01.

The potential of THTMP causing apoptosis in GBM cells was analysed based on the differences in plasma membrane integrity and permeability using Annexin V/PI dual staining. Figure 3A shows the percentage of apoptotic and necrotic cells in THTMP/TMZ treatment in GSC and NSCC of LN229 and SNB19 cells. Exposure of GSC and NSCC of LN229 and SNB19 with THTMP/TMZ induced the apoptosis and necrosis. NSCCs have a greater percentage of apoptotic cells compared to GSCs in both cell lines, in agreement with the cytotoxicity results. THTMP exposed GSC-SNB19 has higher apoptosis than GSC-LN229, which was about 30% and 10%, respectively. Meanwhile, THTMP treated NSCC population showed 40% of apoptosis in both cell lines, while TMZ showed 25% and 30% apoptosis in LN229 and SNB19, respectively (Figure 3A). This suggests the better potentiality of THTMP to induce apoptosis both in NSCC and GSC population.

To explore the genes involved in inducing the apoptosis on THTMP treatment, gene expression profiling of GSC and NSCC was also performed. GO term enrichment analysis was conducted to detect the biological significance of genes in cell death, programmed cell death and apoptosis process (Supplementary Tables S5–S8). It is figured out that a greater number of DEGs related to apoptotic signaling pathway was enriched in THTMP treated condition than in the control (Figure 3B). Among them, we found that NSCC was modulated more than GSC, which is also evidenced from the previous data of apoptosis assay. It is further validated by observing the upregulation of the pro-apoptotic genes (*BBC3*, *BCL2L11* (*Bim*), *BCL2A1*, *MADGED1*) and as well downregulation of a few pro-survival genes (*BCL2*, *BCL2L1* and *MCL1*).

Notably, *BBC3*, a *p*53 upregulated modulator of apoptosis (*PUMA*) was significantly upregulated in GSC-SNB19. *PUMA* is induced in cells following p53 activation by binding to Bcl-2 and further localizes to mitochondria that induces cytochrome *c* release and finally activates programmed cell death. Thus, in GSC, upregulation of *BCL2A1* and *BCL2L11* was observed, indicating the role of THTMP in p53 inducible apoptosis. In contrast, the upregulation of *BIRC3* which encodes for BIRC protein, a member of the inhibitor of apoptosis gene (IAPs) unfortunately prevents apoptosis in GSC-LN229 and NSCC-LN229 cells. Nevertheless, a strong downregulation of *BIRAC5* was observed in NSCC-SNB19 cells.

Accumulation of reactive oxygen species (ROS) at the mitochondria is one of the apoptotic stimuli in the intrinsic death pathway. High level of ROS might damage proteins, nucleic acids, resulting in oxidative stress and cellular dysfunctions [39,40,41,42]. To study the ROS mediated apoptosis on treatment with THTMP/TMZ, ROS assay was performed using H2DCFDA. THTMP exposed cells showed increased apoptosis due to the higher ROS production which is directly proportional to H2DCFDA-flourescence intensity. THTMP treatment significantly produced higher fold level of ROS with 2.5 and 2.3 in NSCC-LN229 and NSCC-SNB19, respectively. However, 2-fold increase of ROS was noticed in GSC-LN229 while only with 1.2-fold in GSC-SNB19 (Figure 3C). ROS level was found to be higher in THTMP treated cells than in TMZ, DMSO control and H_2_O_2_ control. Furthermore, oxidative gene expression signatures confirmed several DEGs related to ROS such as *FOXM1*, *TXNRD2*, *DUSP1*, and *SOD1* were also enriched (Supplementary Tables S1–S4). These genes are associated with poor patient prognosis in several tumor types including glioblastoma [43,44].

In addition to the ROS mediated apoptosis, we have also analysed the possible role of caspase mediated apoptosis. It is been evident that, there seems to be a least level of caspase 3/7 activation on treatment with both THTMP and TMZ (Figure 3D). Yet, TMZ has highest fold increase in caspase activity than in THTMP. GSC-LN229, GSC-SNB19 has higher caspase activity in TMZ than THTMP treatment. NSCC-LN229 has higher caspase activity than NSCC-SNB19 in TMZ and vice versa in THTMP. This could be a possible reason for higher cytotoxicity in NSCC-SNB19 upon THTMP treatment than other conditions (Figure 1D). This endorse that THTMP could selectively induce apoptosis of GSC and NSCC population of both cell lines via caspase 3/7 activation.

### 3.5. Effect of THTMP on GSC and NSCC Gene Expression

**Figure 4:**
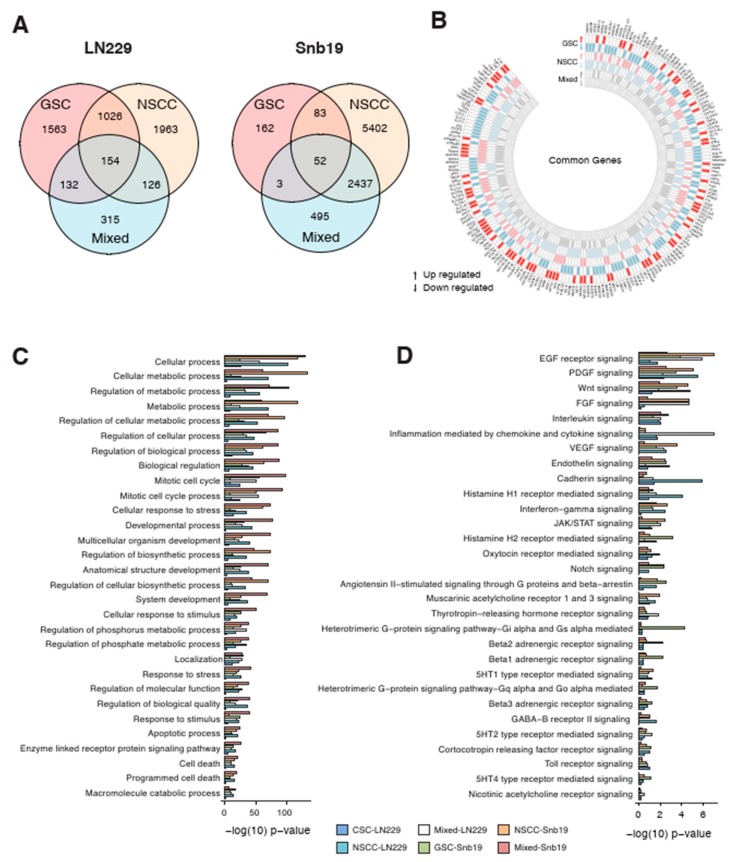
Effect of THTMP on GSC, NSCC and their progenitors gene expression patterns (**A**) Overlapping DEGs in GSC, NSCC and mixed populations of LN229 and SNB19 cell lines. (**B**) Circos plot of overlapping up- and down-regulated genes detected in GSC, NSCC and progenitor lines. (**C**) Common KEGG biological process in GSC, NSCC and progenitor lines. (**D**) Common signaling pathways in GSC, NSCC and progenitor lines.

Global gene expression profiling was performed to determine the mechanism of action of THTMP on GSC and NSCC proliferation and survival. Gene set enrichment analysis explored the DEGs of GSC-LN229 and NSCC-LN229 up to 2875 and 3269 respectively, whereas NSCC-SNB19 and GSC-SNB19 has 7974 and 300 DEGs, respectively (Figure 4A). Genes that were enriched either in a upregulation or downregulation fashion (Figure 4B), have been reported to be highly associated with various biological functions such as cellular process, DNA replication, DNA repair, cell cycle, chromatin remodelling, apoptotic process and programmed cell death (Supplementary Tables S5–S8). We also analysed the DEGs of GSC and NSCC with the progenitor cells, namely mixed-LN229 and mixed-SNB19. It is revealed that there are 154 genes shared in common with GSC, NSCC and mixed population, while only 52 genes in SNB19 (Figure 4A, Supplementary Tables S9 and S10). The least sharing of DEGs could be because of the less number of genes expressed in GSC-SNB19. All these data suggest that a few of the biological processes (Figure 4C) and signaling pathways (Figure 4D) were affected in those populations on treatment with THTMP. The influence of THTMP to modulate the cellular process, metabolic process, mitotic cell cycle, apoptotic process, programmed cell death in all the populations of GBM cells was observed (Figure 4C).

### 3.6. Effect of THTMP on Multiple Signaling Pathways 

**Figure 5:**
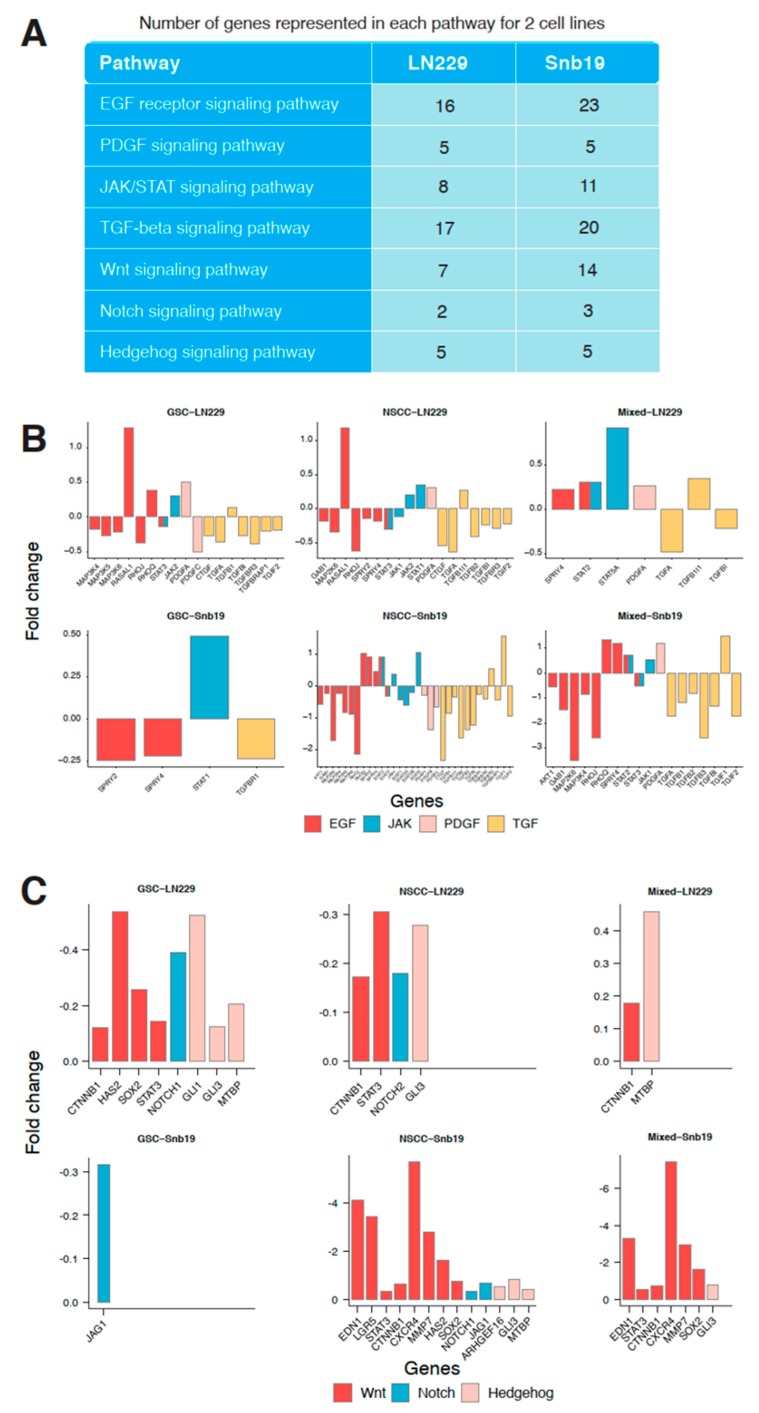
Effect of THTMP on multiple signaling pathways (**A**) Significant targeted signaling pathways upon THTMP treatment. (**B**) Comparison of DEGs fold change in EGF, PDGF, JAK/STAT, and TGF-β signaling pathways of GSC, NSCC and mixed populations. (**C**) Comparison of DEGs fold change in CSC signaling pathways in GSC, NSCC and progenitor lines.

To identify the association of modulated signaling pathways, six main targeted pathways were selected for the further analysis (Figure 5A). The number of genes represented in each pathway for both cell lines was also listed in Figure 5A. The more number of DEGs was associated with EGFR pathway suggests its significant role in modulating the pathway, while the PDGF pathway even with only five DEGs, identified as the second most significant pathway. Various other pathways like JAK/STAT, TGF-beta, Wnt, Notch and Hh pathways also shows significant modulation when the cell lines are treated with THTMP. EGFR signaling plays an important role in promoting glioblastoma survival and progression and thus by targeting EGFR pathway, the cancer cells either will undergo apoptosis or becomes sensitized to chemotherapy [15]. Our analysis provides a prime data on understanding the dysregulated genes in EGFR pathways upon THTMP treatment.

Figure 5B shows the hub genes which are up and down regulated includes *RHOQ*, *SPRY2*, *SPRY4*, *STAT2* and *MAP3K4*, *MAP3K5*, *RHOJ*, *AKT1*, *GAB1* in GSC, NSCC and their progenitor of LN229 and SNB19. More number of genes were regulated in EGFR signaling in GSC and NSCC population of LN229 than the mixed population. Unlike LN229, more number of EGFR signaling genes in SNB19 cells was targeted in GSC, NSCC and mixed population. Targeted EGFR signaling also leads to the interruption of JAK/STAT pathway [45,46,47]. It was also figured out that JAK/STAT signaling of GSC, NSCC and mixed population was modulated under THTMP treatment.

Figure 5B shows that JAK/STAT signaling was highly targeted in NSCC rather than GSC and mixed populations in both cell lines. JAK/STAT was interrupted throughout the dysregulation of *JAK1*, *JAK2*, *STAT1*, *STAT3* and *STAT5A* genes. In addition, the PDGF signaling interruption was also recorded in those populations. In this study, we determined that *PDGFA* gene was upregulated in GSC-LN229, NSCC-LN229 and mixed-LN229 and mixed-SNB19 cells. A fold change of downregulated *PDGFC* gene was also found in GSC-LN229 and NSCC-SNB19 cells. It is observed that even though the PDGF signaling pathway was modulated by THTMP, different gene sets were regulated depending on different characteristics of the cell population and cell line. In addition to altered signaling of growth factors, we also found the interruption of TGF-beta signaling pathway by THTMP. TGF-beta signaling was affected by a wide range of genes such as *CTGF*, *TGFA*, *TGFB2*, *TGFB3*, *TGFBR1*, *TGIF1* and *TGIF2* in all the populations. The pattern shows that the NSCC was highly targeted in this signaling, when compared to the GSC and mixed population in both cell lines.

### 3.7. GSCs and NSCCs Supports Each Other

Transcriptome profiling evidenced that CSC signaling pathways such as Wnt, Notch and Hh was also targeted by THTMP treatment. Figure 5C shows the downregulation of the genes involved in these three signaling pathways. In LN229, both the GSC and NSCC populations were highly modulated than the mixed cells. The similar result was also observed in NSCC-SNB19 while GSC-SNB19 was quite difficult to be targeted than their mixed populations. 

The Wnt signaling pathway was targeted mainly by THTMP in all the cell populations except GSC-SNB19, because of the downregulation of bulk crucial genes, CTNNB1, HAS2, SOX2, STAT3, EDN1, LGR5, CSCR4 and MMP7. To be more specific, *CTNNB1* was down regulated which decipher for β-catenin, thus leading to the inhibition of Wnt signaling. Downregulation of genes such as *GLI1*, *GLI3*, *MTBP* of Hh pathway and *JAG1*, *NOTCH1*, *NOTCH2* of Notch pathway, suggest that THTMP significantly inhibits these pathways in all the cell populations. Notably, Notch signaling pathway was only targeted in GSC-SNB19. Thus, it is evident from the data, separating GBM cell lines into individual GSC and NSCC population, makes them more easily targeted than their mixed cells. These observations revealed that GSC and NSCC could possibly have a connection that benefits each others proliferation and provides resistance to the drugs.

### 3.8. Induction of Cell Death in Patient-Derived GBM Cell Lines by THTMP

**Figure 6:**
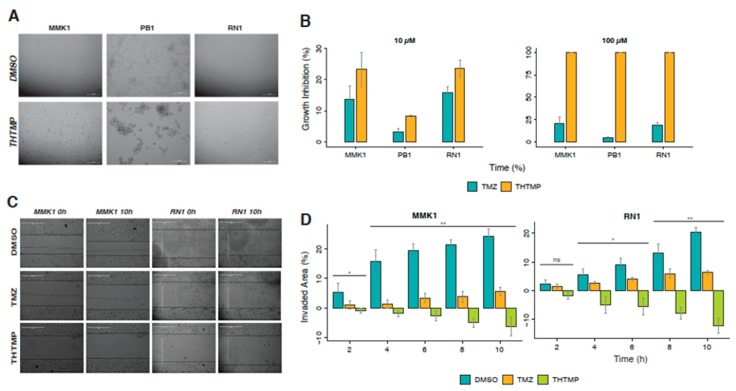
THTMP inhibited cell growth and cell migration of patient-derived mesenchymal subtype of GBM cells. (**A**) Demonstrated images of morphological changes in patient-derived GBM cells at 24 h after THTMP and DMSO treatment. (**B**). Growth inhibitory effect of THTMP on different cell lines, MMK1, RN1 and PB1 at 10 and 100 µM after 24 h post treatment. (**C**) Example images of scratch assay shows the closing/widening of the scratched area over the time. All images were taken using a light microscope with 10× objective. (**D**) Quantification of the percentage of invading area of MMK1 and RN1 cells for every 2 h over the period of 10 h after the scratch. * *p* < 0.05, ** *p* < 0.01, ns—non significant.

Low-passage, serum-free cell lines, MMK1, RN1 and JK2 cultured from mesenchymal patient tumour tissue are used for preclinical study especially for the cell death analysis. The cells were treated with THTMP/TMZ at 10 µM and 100 µM for 24 h. Microscopic observation of GBM patient-derived cell lines revealed the loss of adherence property and changes in the morphology under THTMP at 100 µM (Figure 6A) treatment, remains unaffected in the DMSO treated condition. THTMP strongly inhibited the growth of mesenchymal patient-derived GBM cells compared to TMZ (Figure 6B). Absolute 100% cell death at 100 µM in all of three cell lines was observed upon treatment with THTMP. At 10 µM, the higher growth inhibition was found in MMK1 and RN1 approximately with 24% and 23%, respectively and with only 8% in PB1. Similar patterns of cell death were also observed in 10 µM TMZ treated cells, while around 20% of growth inhibition was observed in MMK1 and RN1 at 100 µM treatment. However, the growth inhibition percentage of TMZ was remarkably lower when compared with THTMP treatment. These results indicate THTMP as the promising agent of an inhibitor of patient-derived GBM cells.

The cell migration and invasion are one of the most important characteristics of malignant tumor cells [48] and indeed, inhibiting cell migration is considered as a potent target for developing new anticancer therapy. Wound healing assay was performed on these three patient-derived mesenchymal cell lines for a period of 10 h upon treatment with THTMP/TMZ. Among these cell lines, PB1 has low adherent ability, and so they are lifted immediately after the scratch and hence the assay was not performed in this particular cell line. The MMK1 and RN1 after THTMP treatment, the invaded areas decreased steadily over time (Figure 6C,D) whilst it increased steadily over time after DMSO and TMZ treatment. After 10 h of post treatment, invaded area of MMK1 cells increased to 20% by DMSO, while only 6% by TMZ and −5% by THTMP. The similar pattern was also observed in RN1. These results confirm that THTMP has the ability to reduce not only the cell proliferation, but also the migration due to enhanced cell death in patient-derived cells, thus further confirming THTMP as a potent drug.

## 4. Discussion

Tumorigenic cancer stem cells (CSCs) is a fraction of sub population of cells present in all tumor involved in invasion, metastasis and relapse. CSC have self-renewal ability and the capacity to become differentiated cells, thus allowing them to create resistance to current radiation and chemotherapy treatment [6]. In recent years, efforts have been made in elucidation on the molecular mechanisms to suppress the specific CSCs [7,8,9,10]. Therefore, many therapeutics have been developed targeting specific signaling pathways in order to inhibit the activation of CSCs [49].

In the present study, we were able to focus on the anticancer property of THTMP against GSC and NSCC population. THTMP inhibits the GSC and NSCC proliferation in a time dependent manner, thereby causing DNA damage, and the cells get arrested at G1/S phase and G2/M phase. Transcriptome analysis confirms the anti-proliferation activity of THTMP on these populations. *FOXM1*, a major hallmark of cancer was significantly downregulated in GSC and NSCC. *FOXM1* is known as an essential transcription factor that is required for various biological process such as DNA damage repair, cell renewal, cell proliferation and cell cycle progress [50]. Thus, THTMP act as a FOXM1 inhibitor thereby reducing the CSC proliferation.

Transcriptome analysis revealed that different set of genes associated with apoptosis was enriched in different populations. For example, pro-apoptotic genes like *BCL2L11* and *BBC3* were upregulated in GSC and NSCC of SNB19 while *BCL2A1* and *MAGED1* were found to be upregulated in GSC and NSCC of LN229. The same pattern was also observed in anti-apoptotic genes in these populations. It’s been noticed that cluster of genes were highly targeted in NSCC than GSC. Therefore, THTMP could induce cell death by regulating several key genes involved in apoptosis process.

A complete understanding on the biological features of CSCs is essential for the development of a new anticancer therapeutics. Among several mechanisms proposed so far on therapeutic resistance in CSCs, we mainly focused on the cell cycle regulation in CSCs. Two approaches have been proposed to prevent recurrence of the tumor. The first approach is known as induction of the entry of CSCs into cell cycles to increase their sensitivity to anticancer therapy (wake up therapy) whereas the second strategy is to forcefully maintain the CSCs dormancy (hibernation therapy), that prevents the generation of new cells [51]. According to the gene expression profile, *Skp2* gene coding for S-Phase Kinase Associated Protein 2 was not modulated. This protein reduces the frequency of aldehyde dehydrogenase positivity among the cancerous cells which are the indicators of CSC function [52]. This data suggests that THTMP could not promote the dormancy of CSCs, in contrast, we could observe that the CXC chemokine receptor 4 signaling (*CXCR4*) is modulated by THTMP, which confirms its role in the wake-up therapeutic strategy in targeting CSCs [53,54].

The gene expression pattern of several signaling pathways such as EGF, PDGF, JAK/STAT, TGF-β were affected upon THTMP treatment. It is known that PDGF signaling regulates tumor growth and metastasis [55,56]. In GBM, the overexpression of *PDGFA* has a retention motif enhancing its autocrine stimulatory effect, was also found to efficiently promote GBM development [57]. THTMP has the ability to modulate PDGF signaling via downregulation of *PDGFA* in selective population of both the cell lines. The transcription factor signal transducer and activator of transcription STAT3, functions as a double-edged sword that behaves both as an oncogene and as well as onco-suppressor. Earlier research in curcumin, a natural phenol, has inhibited cancer stem cells via downregulation of STAT3 [58,59] which is also observed in the cells that were treated with THTMP.

Despite the consistent association of THTMP in targeting various CSC signaling pathway in both GSC and NSCC population, it has been shown to inhibit Wnt, Notch and Hh pathways. The intricacy mechanism of the Wnt signaling pathway provides various levels of therapeutic intervention and the gene expression analysis provides plethora of approaches being discovered to reduce Wnt/β-catenin signaling output [11]. Along with *CTNNB1*, other crucial genes of Wnt pathway were also downregulated. Our data presents the ability of THTMP to drive the downregulation of *LGR5* in NSCC-SNB19 that could help the inhibition of precursor CSC. Also, *EDN1* which is secreted by most of the solid tumors for the persistent growth and survival by suppressing apoptosis [60] is also downregulated in NSCC and mixed-SNB19 cell population. Downregulation of a few significant genes creates a cascade of events in modulation and further inhibition of CSC. THTMP modulates MMP7 regulators and consequently inhibits the invasion and metastasis of CSC in GBM cells. It is observed that Wnt has a wide range of effects on different GBM population except, GSC-SNB19 with similar effect in Hh pathway. On the other hand, Notch pathway has been modulated in CSC signaling pathways on GSC-SNB19 through the down-regulation of *JAG1*. Henceforth, limited modulated genes were enriched in the progenitors, LN229 and SNB19 when compared to the GSC and NSCC populations.

Overall data suggest the significant role of THTMP in cell cycle arrest and apoptosis induction via modulating EGFR and CSC signaling pathway. The ability of THTMP in inhibiting the GBM cell proliferation and the TMZ resistant variants, implies THTMP as a clinically potential agent. The present research reveals that THTMP could also reverse TMZ resistance property in CSC population of GBM cells, by reducing its proliferation and migration. Therefore, THTMP can be considered to develop an adjuvant chemotherapeutic agent for treating GBM.

## 5. Conclusions

The data concluded the potential implications of THTMP in GBM treatment by inducing DNA damage through ROS-mediated apoptosis in GSC and NSCC population. THTMP sensitizes the TMZ variance and thus inhibits the GBM cell growth and proliferation. THTMP plays crucial role in regulating the genes involved in the major check points in the cell cycle pathways. Additionally, THTMP regulates the tumorigenicity of GBM through the modulation of CSC and EGFR signaling pathway. Thus, our present study unravels the new insights of exploiting THTMP in modulating signaling pathways that targets the cell proliferation and migration. Overall, THTMP based adjuvant chemotherapeutic agent can be developed for the GBM treatment.

## Supplementary Materials

The following are available online at https://www.mdpi.com/2073-4409/9/3/681/s1, Table S1: The list shows the genes that were differentially expressed in THTMP vs Untreated in GSC-LN229, Table S2: The list shows the genes that were differentially expressed in THTMP vs Untreated in NSCC-LN229, Table S3: The list shows the genes that were differentially expressed in THTMP vs Untreated in GSC-SNB19, Table S4: The list shows the genes that were differentially expressed in THTMP vs Untreated in NSCC-SNB19, Table S5: The list shows the GO biological processes that were differentially modulated in THTMP vs Untreated in GSC-LN229, Table S6: The list shows the GO biological processes that were differentially modulated in THTMP vs Untreated in NSCC-LN229, Table S7: The list shows the GO biological processes that were differentially modulated in THTMP vs Untreated in GSC-SNB19, Table S8: The list shows the GO biological processes that were differentially modulated in THTMP vs Untreated in NSCC-SNB19, Table S9: The list shows the DEGS that were shared in common with GSC, NSCC and mixed population in LN229, Table S10: The list shows the DEGS that were shared in common with GSC, NSCC and mixed population in SNB19, Figure S1: Growth inhibitory effect of THTMP on different cell lines at 10 μM at 24 h post treatment.

## Abbreviations

GBM	Glioblastoma multiforme
CSC	Cancer stem cells
NSCC	Non-stem cancer cells
GSC	GBM stem cells
TMZ	Temozolomide
ROS	Reactive Oxygen Species
RNA-seq	RNA-sequencing
DMEM	Dulbecco’s Modified Eagle’s Medium
DNA	Deoxyribonucleic acid
FBS	Fetal Bovin Serum
GO	Gene Ontology
DEG	Differentially expressed gene
